# Intention for Warm-Up among Children and Adolescents Scale: Development and Initial Validation

**DOI:** 10.3390/ijerph191711033

**Published:** 2022-09-03

**Authors:** Liyi Ding, Judy L. Van Raalte, Marcia Mackey, Britton W. Brewer, Min Jin, Minming Chu, Lijun Weng

**Affiliations:** 1 Physical Education College, Shanghai Normal University, Shanghai 200234, China; 2 Department of Psychology, Springfield College, Springfield, MA 01109, USA; 3 College of Health Sciences, Wuhan Sports University, Wuhan 430079, China; 4 Department of Physical Education & Sport, Central Michigan University, Mt. Pleasant, MI 48859, USA; 5 Feng Xian Institute of Education, Fengxian District, Shanghai 201499, China

**Keywords:** reliability, construct validity, warm-up, children, adolescents

## Abstract

The purpose of this study was to develop and validate the Intention for Warm-up among Children and Adolescents Scale (IWCAS). There were four phases and four sets of participants in the development of the IWCAS. In the first phase, the domains of intention were defined, and related components were developed, organized, and validated. In the second phase, 446 elementary and middle school students participated in a pilot study for the first version of the scale, which was revised based on the information obtained. In the third phase, 12 graduates in sports pedagogy served on an expert panel and organized the items into domain areas and developed a second version of the IWCAS. In the final phase, 1322 elementary and middle school students from three k-12 schools completed the revised version of the IWCAS, and exploratory and confirmatory factor analyses were conducted. Based on the results, the IWCAS was shortened by deleting some items in two domains; this resulted in 11 items of the final version with 3 domains: (1) attitude toward warm-up, (2) subjective norm, and (3) perceived behavioral control that, according to the indices, generate reliable and structurally valid scores. The composite internal consistency for the three domains ranged from 0.74 to 0.85. The researchers hypothesized the IWCAS is a valid and reliable scale, which can be used by P.E. teachers or coaches to evaluate the intention of primary and secondary school students to perform warm-ups.

## 1. Introduction

Participation in sport has many advantages for children and adolescents, including reducing cardiovascular risk factors, enhancing bone health, and decreasing obesity and Type 2 diabetes [1,2]. However, participation in sport also comes with an inherent risk of sport-related injuries [3,4]. It is estimated that 20% of schoolchildren are absent from school at least one day a year due to sport injuries, and one in three youths seek medical attention for a sport-related injury annually [5]. The implementation of interventions that reduce sport injuries among children and adolescents could mitigate some of the negative outcomes of sport participation.

Physical warm-ups involve a series of physical exercises of gradually increasing intensity performed before more vigorous exercise and sport activities. Previous intervention studies have shown that warm-ups were effective interventions to prevent sport injuries [6,7,8,9]. For example, LaBella et al. conducted research in the neuromuscular intervention program in 2011. They found that coach-led neuromuscular warm-ups reduced non-contact low extremity injuries in female high school soccer and basketball athletes from a mixed-ethnicity, predominantly low-income, urban population [6]. In 2016, Richmond et al. [7] used a 12-week high-intensity neuromuscular warm-up (including aerobic, strength, balance, and agility components) to reduce the risk of school sports injuries in Canadian secondary school students aged 11–15. Results showed the intervention group had a 70% reduction in the risk of school sports injury, compared to the control group (incidence rate ratio = 0.30, 95% CI 0.19–0.49). A recent meta-analysis indicated that effective physical warm-up intervention programs (WIP) include comprehensive, neuromuscular, and balance programs [10]. Although physical warm-up programs have proven to be efficacious in decreasing sport injuries, it is not clear what factors contribute to their use. In addition, although there have been previous studies on warm-up scales, they have mainly dealt with attitudes toward warm-ups only. Therefore, those results will have a certain limitation on the effect of the final behavior prediction.

The theory of planned behavior (TPB) is one of the most influential behavior prediction theories in psychology. It can be used to explain the process of individual behavioral decision-making [11]. TPB is an extended model derived from the theory of reasoned action (TRA). TPB holds that the purposeful and planned rational behavior of individuals is dominated and influenced by their behavioral intentions. The strength of an individual’s behavioral intention is affected by three factors: behavioral attitude (BA), subjective norm (SN), and perceived behavioral control (PBC) [12]. Behavioral attitude means the degree to which a person has a favorable or unfavorable evaluation of the behavior of interest. Subjective norm is the belief about whether most people approve or disapprove of the behavior. Perceived behavioral control refers to a person’s perception of the ease or difficulty of performing the behavior of interest. Regarding warm-ups for exercise, the TPB suggests that students who hold a positive attitude toward warm-ups, view warm-ups as normative behavior and perceive themselves as having control over engaging in warm-ups and will be likely to intend to engage and actually engage in warm-ups prior to physical activity. Consequently, the purpose of this study was to use the TPB to develop a scale to assess children and adolescents’ intentions to perform physical warm-ups prior to engaging in physical activity.

## 2. Method

The development of the IWCAS consisted of four phases and four sets of participants. Phase 1 focused on defining intention toward warm-ups, generating domains, and developing an item pool for each domain. Phase 2 involved pilot testing the IWCAS with elementary and middle school students from one k-12 school. Phase 3 tested content validity. Experts in sports pedagogy placed each item into its corresponding domain to ensure acceptable expert agreement on the content of each item. In Phase 4, elementary and middle school students from three k-12 schools participated in retesting the reliability and validity of the scores produced by the revised IWCAS.

### 2.1. Definition of Intention, Component Conceptualization, and Item Development (Phase 1)

The purpose of Phase 1 was to develop, revise, and refine items for the IWCAS. In developing the IWCAS, we defined key constructs in line with the TPB [11]. We defined attitude as children and adolescents’ perception of warm-ups, such as the advantages or benefits of warm-ups. We define subjective norms as external pressures, such as those from peers, parents, or teachers that affect children and adolescents’ likelihood of doing a warm-up. Perceived behavioral control was defined as children and adolescents’ self-confidence in doing warm-ups, such as their ability or capability to do warm-ups.

Following a comprehensive review of literature on warm-up programs [13,14], three full-time teachers developed a 37-item pool (Appendix A) that included a priori categories derived from the TPB: attitude (14 items), subjective norm (15 items), and perceived behavioral control (8 items) [12]. Each item was scored on a Likert-type scale ranging from 1 (strongly disagree) and 2 (disagree), to 4 (agree) and 5 (strongly agree). A midpoint score of 3 stood for neutral (neither agree nor disagree).

### 2.2. Pilot Testing the IWCAS (Phase 2)

#### 2.2.1. Participants

Participants for the pilot study were elementary and middle school students (*n* = 446) from one k-12 school in Shanghai with an average age of 13.62 (*SD* = 2.62) years. There were more male students (*n* = 242, 54%) than female students (*n* = 204, 46%). Most participants (*n* = 321, 72%) were middle school students; the rest (*n* = 125, 28%) were elementary school students. 

#### 2.2.2. Procedure

Leaders from the Educational Administration Institution of Shanghai and the k-12 school gave approval for us to conduct the study. Shanghai Normal University’s ethics committee for the protection of human participants approved the study, and all the students and their parents signed an informed consent form, indicating their consent to participate in the research. After all school approvals and consents were granted, the researchers handed out the questionnaire and gave a brief explanation of the questions. They were available to answer questions while the children completed the questionnaire on site. The questionnaire included demographic information, items about school sport injuries in the last 12 months, and the 37-item IWCAS (Appendix A). A total of 560 completed questionnaires were collected, but there were 446 valid questionnaires after deleting questionnaires with missing values and false responses, resulting in a valid return rate of 80%. 

### 2.3. Content-Validity Study (Phase 3)

The Intention for Warmup among Children and Adolescents Scale (IWCAS) was developed using content analysis methods where a set of 20 items (second version) was drawn from the theoretical framework of the TPB [12] after the first version was reviewed by a panel of judges for item-content related validity [15,16]. Results were evaluated from a quantitative and qualitative perspective, as suggested by Dunn et al. (1999) [16]. Expert feedback was used to revise the items of IWAS after conducting a statistical analysis of the first version of IWCAS. We only pooled relevant items from previous studies on the topic and then generated new items based on a conceptual model [17]. Although it was determined in advance to use the TPB to design the items and the structure of the three domains, the domains had not been defined clearly, resulting in the inconsistency across all the items of each domain. For example, in the domain of attitude, item 4 (“I feel I can prevent sports injuries better after warm-ups.”) and item 7 (“I think warm-ups can improve stamina.”) indicated the benefits of warm-ups, but item 13 (“If I had options, I would choose to do warm-ups before physical exercises.”) pertained to the intention to perform warm-ups. Therefore, we redefined the domains, reduced the number of corresponding items for each domain, and produced the second version of the 20-item IWCAS.

#### 2.3.1. Participants

Experts (*n* = 22) currently working at elementary or middle schools as P.E. teachers with a master’s degree in sport pedagogy were invited to participate in the study. All the experts signed a consent form before completing the questionnaire. After deleting the questionnaires with missing responses to one or more of the items, an analysis of 12 valid questionnaires was conducted to determine item content-related validity.

#### 2.3.2. Procedure

The establishment of validity in terms of content-related evidence was achieved when the factors were reviewed by experts. Reviews of statements considered relevance, clarity, and representation of the construct across the content. [15]. After receiving permission to conduct the study from Shanghai Normal University Ethics Committee for the protection of human participants, experts in a wechat group were invited to participate in the research. Interested participants gave their informed consent via a digital informed-consent letter and were then sent a link to the online survey site (Wjx.cn)(accessed on 1 December 2020). The online questionnaire consisted of 3 parts: demographic information, factors/domains definition, and the items and their corresponding domain. Random order was incorporated for the distribution of content to the experts. Experts used a 5-point Likert scale with a range of 1 (poor association) to 5 (excellent association) between each item and the subscale definitions The experts were also asked to provide additional comments after rating the fit of each item in the intended domains.

#### 2.3.3. Statistical Analysis

The ratings of the experts were analyzed through the use of a content validity coefficient (Aiken’s V) [18], which determined if each item significantly loaded onto the keyed domain. Aiken’s V coefficients range from 0 to 1, with values closer to 1 being indicative of higher levels of agreement among the experts. The critical value for Aiken’s V is based on the number of reviewers and number of hypothesized factors (k = 3). For 12 reviewers, Aiken’s V coefficient of greater than 0.69 was significant at the 0.05 level [18].

Cohen’s d [19] was calculated to determine if the rating of each item were too closely related to a non-intended domain. Cohen’s ds greater than 0.80 were considered large, indicating the item did not double-load across factors. Items with medium effect sizes (0.3–0.8) were reviewed with caution for double loading. Any item that had a small effect size (<0.3) was considered to load onto more than one factor [20]. See Appendix C for the formulas used to calculate Aiken’s V and the Cohen’s d. 

Open-ended responses were reviewed with a qualitative examination [16]. The response analysis took into consideration terminology, relevance of content, and clarity, in order to determine any need for item adjustment [16].

### 2.4. Reliability and Validity Study (Phase 4)

#### 2.4.1. Participants

Participants for the study were elementary and middle school students (*n*= 1322) from three k-12 schools in Shanghai with an average age of 12.10 (*SD* = 1.85) years. There were more male students *(n* = 693, 52%) than female students (*n* = 629, 48%). Of the 1322 participants, 26% (*n* = 337) were grade four students, 27% (*n* = 351) were grade five students, 16% (*n* = 210) were grade seven students, 16% (*n* = 208) were grade eight students, and 16% (*n* = 216) were grade nine students. We choose the sample used for exploratory factor analysis from School B (*n* = 334) and the sample for confirmatory factor analysis from schools A (*n* = 374) and C (*n* = 988).

#### 2.4.2. Procedure

The leaders from the Educational Administration Institute of Shanghai and the investigated schools were contacted and permitted us to conduct the survey. After getting their permission and receiving permission to conduct the study from the Shanghai Normal University Ethics Committee for the protection of human participants, the study began. Researchers handed out the second version of the questionnaires in the classrooms. Students were required to sign the informed consent before filling in the questionnaires. Researchers were available to answer students’ questions on site. The second version of the questionnaire included demographic information, an investigation of school sport injury in the last 12 months, and the 20-item IWCAS (Appendix B). Of the 507 completed questionnaires from School A, 374 were complete and valid, indicating a valid return rate of 74%. Incomplete questionnaires and those with false responses were not included. Of the 419 questionnaires from School B, there were 334 valid questionnaires (valid return rate = 80%). From School C, 869 questionnaires were collected, and there were 614 valid questionnaires (valid return rate = 71%).

#### 2.4.3. Statistical Analysis

An EFA was employed to identify any underlying concealed factors of the IWCAS [21,22]. The suppositional structure of the IWCAS was entered into the EFA. Using the SPSS 22.0 (SPSS Inc., Chicago, IL, USA), an EFA was computed using a principal component factor extraction method with varimax rotation [21,22]. The criterion for inclusion was set with a minimum loading factor of 0.40 [23]. Items that loaded on two factors were either restated or removed. The factor structure was decided by scrutinizing the eigenvalues (1 or greater) and a scree plot. [23]. All decisions to formulate a factor structure and add, modify, or remove items were made in view of the theoretical framework of the TPB [12].

After completing the EFA in Phase 2, the predetermined factor structure was tested using a confirmatory factor analysis (CFA). A priori alternative one-factor solution was tested as well. Additional models based on modification indices were also scrutinized for fit. AMOS 23.0 (SPSS Inc., Chicago, IL, USA) was used to conduct the CFA. Chi-square (χ^2^) goodness-of-fit and degrees of freedom were reported for the models. Changes in χ^2^ and df for the alternate models were reported. Meanwhile, both absolute and incremental fit indices were reported. According to Hu and Bentler (1999) [24] and Byrne (2001) [25], the following cut-off criteria were used for each of the fit indices: standardized root mean residual (SRMR < 0.06), root mean square error of approximation (RMSEA < 0.08), comparative fit index (CFI > 0.90), and Tucker–Lewis index (TLI > 0.90). Akaike Information Criterion (AIC) and ∆ꭓ^2^ were used to compare models, and lower AIC and ꭓ^2^ values were indicative of improved fit. Standardized regression coefficient weights (Lambda X values) of each item were calculated. The TPB [12] was also considered when deciding to modify the factor structure or scale items. Composite reliability [26] was calculated for the resulting domains of the accepted factor solution.

## 3. Results

The results of the study are presented by the phases of the IWCAS development. First, the results from the Phase 3 content validity study are presented. Then, the phase 4 exploratory factor analysis results are presented; and, finally, the findings from the phase 4 confirmatory factor analysis are reported.

### 3.1. Content Validity (Phase 3)

Items with significant Aiken’s V and Cohen’s d ES above 0.40 remained. See Table 1 for average Aiken V scores for each domain. Sixteen of the twenty initial items rated were kept without any modifications. One item (Item 15) was deleted due to a small ES value (0.16). Despite having an acceptable Aiken’s V, items #10, #11, and #12 were rewritten as a result of a small Cohen’s d value for each item (−0.09, 0.00, and −0.08, respectively) from the scores of the experts’ panel. For example, the item “my parents think I need to do warm-ups well before exercising” had no obvious external influence and was rewritten as “if my parents tell me to do warm-ups before exercise, I will do as they said.”

### 3.2. EFA (Phase 4)

A principal component factor extraction method with varimax rotation was adopted in exploratory factor analysis (EFA). A 4-factor solution emerged after examining the scree plot at the rotated eigenvalues, explaining 56% of the variance in the model. Eigenvalues ranged from 1.10 to 7.01 in Table 2. Table 2 contains the factor loading for the various items. 

Factor 1, explaining 37% of the variance in the model, contained items proposed to be part of the subjective norm (*n* = 4) and perceived behavioral control (*n* = 3) domains. From the seven items that loaded onto the first factor, two items were removed due to double loading. Item 16 originally proposed for the perceived behavioral control domain was removed as well because the item loaded on the factor of the subjective norm. Thus, the first factor retained four items and was named as subjective norm.

Factor 2 consisted of four attitude items, explaining 7% of the variance in the model. The four items loaded exactly on the proposed domain of attitude, and we retained all of them for further analysis. Because the four selected items were all related to injury prevention, the second factor was named as a warm-up attitude toward injury prevention.

Factor 3 consisted of five perceived behavioral control items, explaining 6% of the discrepancy within the model. The five items were all loaded onto the proposed domain of perceived behavioral control. Two items, however, were removed because of double loading on the domains of subjective norm and perceived behavioral control, respectively. The third factor was finalized with three items and was named as perceived behavior control.

Factor 4 consists of four items regarding attitude and represented 6% of the variance. They were, however, separated from the four items on factor 2. According to the content of the items, the fourth factor was named as attitude toward warm-up effects on the human body.

In the exploratory factor analysis, Item 5 did not align with any factor and was, therefore, removed from subsequent analyses. Eleven items were retained for the second version of the IWCAS among the remaining nineteen items. The items encompassed a 3-factor model to be confirmed further via a CFA.

### 3.3. CFA (Phase 4)

As for the results of a CFA, the χ^2^ statistic; CFI; TLI; SRMR; RMSEA; and the standardized regression factor loadings (Lambda X) values are considered some important indicators to determine the fit of the model. A summary of the χ^2^ analysis and fit indices in the three tested models is provided (Table 3). The χ^2^ statistics for the three models were all significant (*p* < 0.05), which indicated a difference existed between the implied covariance matrix and the sample covariance matrix. Several researchers have reported over-sensitivity of the χ^2^ statistic with non-normal data and large sample sizes [24,25]. As a result, other fit analyses were evaluated.

The four-factor solution from the EFA was tested (M2). The fit indices were acceptable (CFI = 0.972, TLI = 0.965, SRMR = 0.032, and RMSEA = 0.045). When examining the factor loadings, all the items had acceptable standardized regression coefficients ranging from 0.59 to 0.86. A comparison to the single-factor model (M1) was made. The fit of the model became worse. The χ^2^ change was significant, M2 was significantly better than the one-factor model solution. Therefore, the lack of fit for M1 indicates that the IWCAS should have an underlying factor structure.

The χ^2^ change in the third model (M3: ∆χ^2^ = 127.01, ∆df = 43) was significant (*p* < 0.05), compared to M2, indicating improved model fit. All the fit indices were acceptable (CFI = 0.980, TLI = 0.973, SRMR = 0.03; and RMSEA = 0.046). The factor loadings of the 11 items in M3 ranged from 0.62 to 0.81. A summary of the fit statistics for all the models is presented in Table 3. Appendix D contains the 11-item scale for M3.

**Figure 1:**
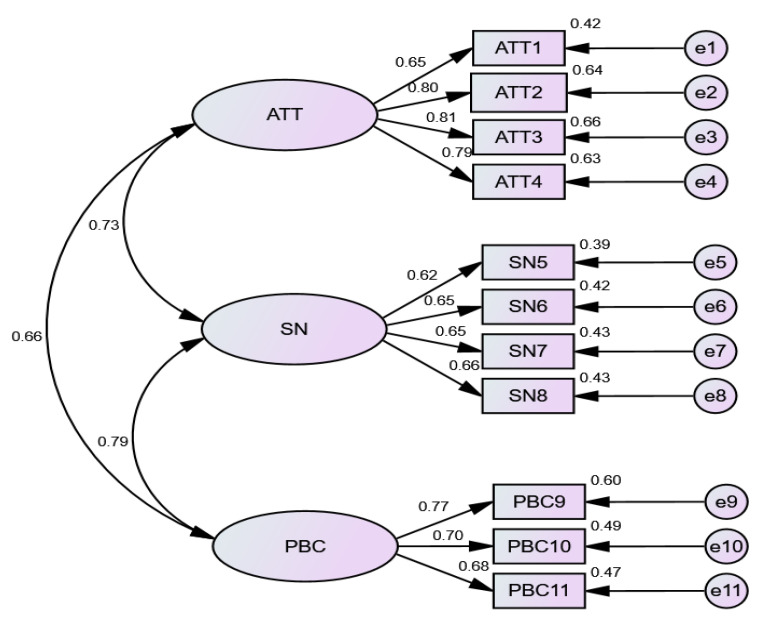
Final three-factor model of IWCAS (Intention for Warm-up among Children & Adolescents Scale).

The means, standard deviations, and correlations of the three subscales derived from the accepted three-factor solution are presented in Table 4. Weighted omega [26] composite reliability for each of the three domains ranged from 0.74 (subjective norm) to 0.85 (attitude). Domains with composite reliability greater than 0.70 were considered to be acceptable levels of internal consistency [26,27]. The remaining three factors were moderately to strongly correlated, with phi coefficients ranging from 0.53 to 0.60. The accepted model of the IWCAS is presented in Figure 1.

## 4. Discussion

Several metrics have been used to determine how well the data fit a particular model [28,29,30]. Construct validity was determined by using four measures (SRMR, RMSEA, CFI, and TLI), which were selected in accordance with current common practice in psychometrics [24,25]. Some previous studies have also used these metrics to test construct validity [20,31].

In this study, the participant to item ratio was close to 90:1, well above the 10:1 ratio recommended for CFA [27]. Scores for the 11 IWCAS items showed acceptable reliability and validity. Internal consistency statistics for each dimension of the IWCAS scale indicated that IWCAS produced reliable scores (i.e., omega greater than 0.70). The construct validity indexes were all within the acceptable range. The CFI is close to 1.00, indicating an excellent fit.

The content validity of the IWCAS scale was judged by graduate level students in the field of physical education. These graduate students are considered experts in the field of IWCAS design because of their professional training and experience in teaching physical education. Although there is no widely accepted standard for the minimum number of experts required to assess the effectiveness of content, it is common practice to survey a panel of 5–20 people to ensure the validity of the content. In the current study, 12 experts reviewed item content.

The accepted standard level for keeping items in a particular dimension without modification is uncertain [32]. According to Dunn (1999) [16], the size of Cohen’s d effect size, as well as the statistical significance of Aiken’s V statistic, creates the foundation for content correlation of the various items. In the current study, the expert’s Aiken’s V value for retained items was greater than 0.69, and the Cohen’s d coefficient for retained items was greater than 0.4. Items that did not meet these standards were modified or deleted.

Although no studies have shown that content validity research must be done before reliability and validity research begins, it can be useful to conduct content validity research before reliability and validity research. The reason for assessing content validity first is that if some items may not have acceptable content validity (i.e., Aiken’s V is greater than 0.69) and, therefore, should be deleted or replaced. On the other hand, it is impractical to add new items to the scale after the content validity research is completed because another content validity study is required to test the newly added items. Therefore, it is common practice not to add any new items to reliability and validity studies unless absolutely necessary [33]. Therefore, in this study, the content validity of the IWCAS scale was tested first, and then the reliability and validity were tested.

The present study tested the reliability and validity of the IWCAS scale in primary and secondary school students in Shanghai, China. Future research can help determine if the scale can be applied to other countries or regions with different national, economic, and social systems. Future research with young children can help determine if those under the age of 8 years old have sufficient reading comprehension ability to complete the IWCAS.

Recently, according to telephone interviews with primary and middle school PE teachers in China and the United States, we learned that, in the United States, different states and schools have different requirements for primary and secondary PE classes. Some schools have only one physical education class per week, and some schools have 3–4 physical education classes per week; some schools have 30 min of physical education, and some schools have 45 min of physical education; there are warm-ups in P.E. classes in some schools but not necessarily in all schools across the country. In China, the situation of physical education in primary and secondary schools is different from that in the United States. The duration of physical education in primary and secondary schools in China is usually about 45 min. Of course, due to regional differences, it may vary. For example, in Shanghai, the duration of physical education in primary schools is 35 min, and the duration of physical education in middle schools is 40 min. There are at least 2–3 physical education classes per week; in China, all physical education classes have warm-ups, and the duration of warm-ups is typically about 5 min. It is not hard to see that there is a big difference between the two countries through the comparison of physical education classes in primary and secondary schools. Therefore, when the IWCAS scale is used to measure the intention of warm-ups among primary and secondary school students in China and the United States, there will be a difference on the scores of the IWCAS. This also provides a theoretical basis and reference for primary and secondary school students in different countries to participate in different warm-up intervention programs.

Scores on the IWCAS were based on self-reported results, and we did not verify the actual warm-up behavior of primary and secondary school students. Future research should be conducted to determine to what extent IWCAS scores are related to actual warm-up behaviors. In addition, scale development is a dynamic and on-going process, which means researchers should continuously examine the psychometric properties of instruments and provide empirical evidence of reliability and validity. Future researchers should also verify whether evidence of test–retest reliability and criterion-related validity exists.

## 5. Conclusions

The purpose of this research was to develop the IWCAS in a manner that incorporated evaluation of content validity and reliability of the tool. The results of the four phases of this study suggest that the scores produced by the IWCAS were reliable and valid in primary and secondary school students. The IWCAS can be used by P.E. teachers or coaches to understand the intention of primary and secondary school students to perform warm-ups. Understanding the level of students’ warm-up engagement can help P.E. teachers and coaches to better implement warm-ups before exercise among those participants. It can help P.E. teachers or coaches to assess changes in intentions to engage in warm-ups among primary and secondary school students to facilitate the evaluation of the effect of warm-up intervention programs. The relationship between intentions towards warm-ups and sports injuries among primary and secondary school students can also be explored with the aid of the scale. P.E. teachers or coaches can use the IWCAS to develop warm-up intervention programs based on the TPB. Finally, the IWCAS can also be used to analyze the factors associated with the intention to engage in warm-ups in primary and secondary school students.

## Figures and Tables

**Table 1:** Descriptive statistics of Aiken’s V coefficient from experts (*N* = 12).

Subscale	M	SD	Min.	Max.
Attitude	0.86	0.04	0.77	0.88
SN	0.79	0.03	0.75	0.81
PBC	0.76	0.05	0.69	0.85

**Note.** V (0.05) = 0.69; SN = subjective norm; PBC = perceived behavioral control; 20 items.

**Table 2:** Summary of principle-component factor extraction with varimax rotation (*N* = 334).

Factor	Eigenvalue	% of Variance		Cumulative %	
1	7.01	36.88		36.88	
2	1.30	6.82		43.70	
3	1.17	6.16		49.85	
4	1.10	5.77		55.63	
Factor loading					
Item	Proposed Factor	*Varimax Rotation Rotated Factor Loadings*			
		*1*	*2*	*3*	*4*
1	Attitude		0.631		
2	Attitude		0.699		
3	Attitude		0.758		
4	Attitude		0.770		
5 *	Attitude				
6	Attitude				0.554
7	Attitude				0.643
8	Attitude				0.773
9	Attitude				0.545
10	Subjective Norm	0.627			
11	Subjective Norm	0.660			
12	Subjective Norm	0.727			
13	Subjective Norm	0.564			
14	Perceived Behavioral Control	0.588		0.401	
16	Perceived Behavioral Control	0.499			
17	Perceived Behavioral Control	0.406		0.524	
18	Perceived Behavioral Control			0.644	
19	Perceived Behavioral Control			0.783	
20	Perceived Behavioral Control			0.658	

* Note. 1. Kaiser-Meyer-Olkin(KMO) = 0.919, *p* = 0.000 < 0.001; 2. Item 5 was deleted because it did not load on any domain in the scale.

**Table 3:** Summary of χ^2^ and fit indices for alternate models.

Model	ꭓ^2^	df	∆ꭓ^2^	∆df	SRMR	RMSEA	CFI	TLI	AIC
M1	638.05	44	-	-	0.0662	0.117	0.858	0.823	704.05
M2	251.83	84	386.22	40	0.032	0.045	0.972	0.965	353.83
M3	124.82	41	127.01	43	0.03	0.046	0.980	0.973	196.82

**Note.** M1 = one-factor model of intention; M2 = 15-item four-factor model of intention; M3 = 11-item three-factor model of intention; df = degree of freedom; SRMR= Standardized Root Mean Residual; RMSEA= Root Mean Square Error of Approximation; CFI = Comparative Fit Index; TLI = Tucker–Lewis index; AIC = Akaike Information Criterion.

**Table 4:** Domain mean, composite reliability, and phi matrix of the 3-factor IWCAS (11 Items).

Domain (# of Items)	M	SD	OMEGA	1	2	3
1. Attitude (4)	4.51	0.68	0.85	-	0.58 **	0.53 **
2. Subjective Norm (4)	4.46	0.64	0.74		-	0.60 **
3. Perceived Behavioral Control (3)	4.31	0.75	0.76			-

**Note.** Omega = weighted omega composite reliability; ** *p* < 0.001.

## Data Availability

The data presented in this study are available on request from the correponding author.

## Appendix A. First Version of the IWCAS (37 Items)

**Domain 1. Attitude (14 Items)**

1. I think warm-up is essential to prevent sports injuries.
2. I think it is important for our school sports team members to do warm-ups before the start of the game.
3. I don’t think I’ll be at my best when doing movements unless I performed warm-ups beforehand.
4. I feel I can prevent sports injuries better after warm-ups.
5. Both professional and amateur athletes need to do warm-ups before exercises.
6. If I don’t do warm-ups for intense workouts, I’m likely to suffer muscle cramps.
7. I think warm-ups can improve stamina.
8. I don’t try to throw a solid ball with all my strength without warm-ups.
9. I feel more confident in my athletic ability after warm-ups.
10. By warm-ups, my muscles gradually adapt to strenuous exercise
11. My movements seem to be more coordinated after doing prep activities
12. I love doing warm-ups because it helps “relax” my muscles
13. If I had options, I would choose to do warm-ups before physical exercises.
14. If performing warm-ups before strenuous exercise, it would be less likely to get injured.

**Domain 2. Subjective Norm (15 Items)**

15. If everyone is doing warm-ups, I will do it, too.
16. If everyone does warm-ups conscientiously, I will be as well.
17. I do warm-ups only if the teacher monitors me.
18. No matter what others do or not, I will do the warm-ups conscientiously.
19. My parents think I should do warm-ups before exercise.
20. My parents think that in order to prevent sports injuries, I should do warm-ups.
21. When exercising with my parents, they always lead me to do warm-ups.
22. My parents think I need to do warm-ups well before exercising.
23. My parents think I don’t need to do warm-ups before exercising.
24. I have to ask the teacher to lead me to do warm-ups before I do it.
25. Teachers often tell us about the benefits of warm-ups.
26. The teacher told us that in order to prevent sports injuries, we should do warm-ups before sports.
27. Teachers always ask us to do warm-ups before exercise.
28. The teacher does not pay much attention to the warm-ups.
29. I only do it when my best friend is doing warm-ups.

**Domain 3. Perceived Behavioral Control (8 items)**

30. I think I can do warm-ups even if it’s hot or cold outside.
31. I think I can do warm-ups adequately.
32. I think I can get my classmates to do the warm-ups with me.
33. I think I can get my teacher to agree with the warm-ups I do.
34. I think I can prevent sports injuries by warm-ups.
35. I think I can remember doing warm-ups before any exercise.
36. I think I have the knowledge in warm-ups.
37. I think I know how to do warm-ups even if no one demonstrates.

## Appendix B. Second Version of the IWCAS (20 Items)

**Domain 1. Attitude (9 Items)**

1. I think warm-ups can make me perform better when doing movements.
2. I think warm-ups can help me better prevent sprains.
3. I think warm-ups before strenuous exercise can prevent muscle cramps.
4. I think warm-ups can prevent muscle strain during exercise.
5. I think that only after warm-ups, I dare to throw a solid ball with all my strength.
6. I think warm-ups can make my body adapt to strenuous exercise.
7. I think that after warm-ups, I can do more coordinated movements.
8. I think warm-ups help “relax” my muscles.
9. I think warm-ups before exercise can help me quickly get into the state of exercise.

**Domain 2. Subjective Norm (4 items)**

10. I will follow the teacher’s demonstration of warm-ups.
11. If my parents tell me to do warm-ups before exercise, I will do as they say.
12. Our P.E. teacher attaches great importance to warm-up, so he will let us do warm-up before each physical education class.
13. When my good friends are doing warm-ups, I will also be affected by them and do warm-ups together.

**Domain 3. Perceived Behavioral Control (7 items)**

14. I think I can do warm-ups well.
15. I think I can let my classmates do warm-ups with me.
16. I think I can let my teacher identify with the warm-ups I did.
17. I think I can prevent sports injuries by warm-ups.
18. I think I can remember doing warm-up before exercise.
19. I think I have the knowledge about warm-ups.
20. I think even if no one demonstrates warm-ups, I know how to do warm-ups.

## Appendix C. Formulas Used to Calculate Aiken’s V and ES

Aiken’s V (Aiken 1985) [18]:

Aiken’s V = S/[n (c − 1)]

where:
“The values of s for all raters (or items) are then added across the n raters or m items to yield S” (Aiken 1985 [18], p. 133).

s = r − lo
r = expert judge’s rating
lo = lowest response option
n = number of raters
c = highest response option

Cohen’s ES (Dunn et al. 1999 [16], p. 36):

$$ES=\frac{\bar{X}-\bar{Y}}{\sqrt{\sigma_{x}{}^{2}+\sigma_{y}{}^{2}+2r_{xy}\sigma_{x}\sigma_{y}}}$$

where

$\bar{X}$ is the mean rating for varibale X,
$\bar{Y}$ is the mean rating for varibale Y,
$\sigma_{x}{}^{2}$ is the variance of variable X,
$\sigma_{y}{}^{2}$ is the variance of variable Y, and
$r_{xy}\sigma_{x}\sigma_{y}$ is the covariance of X.Y.

## Appendix D. Final Version of the IWCAS (11 Items)

**Domain 1. Attitude (4 Items)**

1. I think warm-ups can make me perform better when doing movements.
2. I think warm-ups can help me better prevent sprains.
3. I think warm-up activities before strenuous exercise can prevent muscle cramps.
4. I think warm-up activities can prevent muscle strain during exercise.

**Domain 2. Subjective Norm (4 items)**

5. I will follow the teacher’s demonstration of warm-up.
6. If my parents tell me to do warm-ups before exercise, I will do as they say.
7. Our P.E. teacher attaches great importance to warm-ups, so he will let us do warm-ups before each physical education class.
8. When my good friends are doing warm-ups, I will also be affected by them and do warm-ups together.

**Domain 3. Perceived Behavioral Control (3 items)**

9. I think I can remember doing warm-ups before exercise.
10. I think I have knowledge about warm-ups.
11. I think even if no one demonstrates warm-ups, I know how to do warm-ups.

## References

[1] Collard D.C., Verhagen E.A., Chinapaw M.J., Knol D.L., van Mechelen W. (2010). Effectiveness of a school-based physical activity injury prevention program: A cluster randomized controlled trial. Arch. Pediatr. Adolesc. Med..

[2] Hallal P.C., Victora C.G., Azevedo M.R., Wells J.C.K. (2006). Adolescent physical activity and health: A systematic review. Sports Med..

[3] Black A.M., Meeuwisse D.W., Eliason P.H., Hagel B.E., Emery C.A. (2021). Sport participation and injury rates in high school students: A Canadian survey of 2029 adolescents. J. Saf. Res..

[4] Ding L., Brewer B.W., Mackey M., Cai H., Zhang J., Song Y., Cai Q. (2022). Factors Associated with School Sports Injury among Elementary and Middle School Students in Shanghai, China. Int. J. Environ. Res. Public Health.

[5] Emery C.A., Pasanen K. (2019). Current trends in sport injury prevention. Best Pract. Res. Clin. Rheumatol..

[6] LaBella C.R., Huxford M.R., Grissom J., Kim K.Y., Peng J., Christoffel K.K. (2011). Effect of neuromuscular warm-up on injuries in female soccer and basketball athletes in urban public high schools: Cluster randomized controlled trial. Arch. Pediatr. Adolesc. Med..

[7] Richmond S.A., Jian K., Doyle-Baker P.K., Nettel-Aguirre A., Emery C.A. (2016). A School-Based Injury Prevention Program to Reduce Sport Injury Risk and Improve Healthy Outcomes in Youth: A Pilot Cluster-Randomized Controlled Trial. Clin. J. Sport Med..

[8] Soligard T., Myklebust G., Steffen K., Holme I., Silvers H., Bizzini M., Junge A., Dvorak J., Bahr R., Andersen T.E. (2008). Comprehensive warm-up programme to prevent injuries in young female footballers: Cluster randomised controlled trial. BMJ Clin. Res. Ed..

[9] Emery C.A., van den Berg C., Richmond S.A., Palacios-Derflingher L., McKay C.D., Doyle-Baker P.K., McKinlay M., Toomey C.M., Nettel-Aguirre A., Verhagen E. (2019). Implementing a junior high school-based programme to reduce sports injuries through neuromuscular training (iSPRINT): A cluster randomised controlled trial (RCT). Br. J. Sports Med..

[10] Ding L., Luo J., Smith D.M., Mackey M., Fu H., Davis M., Hu Y. (2022). Effectiveness of Warm-Up Intervention Programs to Prevent Sports Injuries among Children and Adolescents: A Systematic Review and Meta-Analysis. Int. J. Environ. Res. Public Health.

[11] Ajzen I., Kuhl J., Beckmann J. (1985). From Intentions to Actions: A Theory of Planned Behavior. Action Control: From Cognition to Behavior.

[12] Ajzen I. (1991). The Theory of Planned Behavior. Organ. Behav. Hum. Decis. Process..

[13] Ehlert A., Wilson P.B. (2019). A Systematic Review of Golf Warm-ups: Behaviors, Injury, and Performance. J. Strength Cond. Res..

[14] Smith J.L., Bozymowski M.F. (1965). Effect of Attitude toward Warm-Ups on Motor-Performance. Res. Q..

[15] DeVellis R.F. (2017). Scale Development: Theory and Applications.

[16] Dunn J.G., Bouffard M., Rogers W.T. (1999). Assessing item content-relevance in sport psychology scale-construction research: Issues and recommendations. Meas. Phys. Educ. Exerc. Sci..

[17] Babbie E. (1990). Survey Research Methods.

[18] Aiken L.R. (1985). Three coefficients for analyzing the reliability and validity of ratings. Educ. Psychol. Meas..

[19] Cohen J. (1988). Statistical Power Analysis for the Behavioral Sciences.

[20] Mullin E.M., Leone J.E., Margolis G. (2020). Heterosexist Attitudes in Sport—Gay Men Scale: Development and Initial Validation. J. Sport Behav..

[21] Pedhazur E.J., Schmelkin L.P. (2013). Measurement, Design, and Analysis: An Integrated Approach.

[22] Warner R.M. (2008). Applied Statistics: From Bivariate through Multivariate Techniques.

[23] Stevens J.P. (2012). Applied Multivariate Statistics for the Social Sciences.

[24] Hu L.t., Bentler P.M. (1999). Cutoff criteria for fit indexes in covariance structure analysis: Conventional criteria versus new alternatives. Struct. Equ. Model. Multidiscip. J..

[25] Byrne B.M. (2001). Structural Equation Modeling with AMOS: Basic Concepts, Applications, and Programming.

[26] Allen M.P. (1973). Construction of composite measures by the canonical-factor-regression method. Sociol. Methodol..

[27] Nunnally J.C., Bernstein I.H. (1994). Psychometric Theory.

[28] Browne M.W., Cudeck R., Bollen K.A., Long J.S. (1993). Alternative ways of assessing model fit. Testing Structural Equation Models.

[29] McDonald R.P. (1999). Test Theory: A Unified Treatment.

[30] Schumacker R.E., Lomax R.G. (1996). A Beginner’s Guide to Structural Equation Modeling.

[31] Mullin E.M. (2013). Scale development: Heterosexist attitudes in women’s collegiate athletics. Meas. Phys. Educ. Exerc. Sci..

[32] Gable R.K., Wolf M.B. (1993). Instrument Development in the Affective Domain: Measuring Attitudes and Values in Corporate and School Settings.

[33] Keating X.D., Silverman S., Kulinna P. (2001). The development of an instrument measuring preservice physical education teacher attitudes toward fitness tests in schools. Meas. Phys. Educ. Exerc. Sci..

